# Effects on Serum Hormone Concentrations after a Dietary Phytoestrogen Intervention in Patients with Prostate Cancer: A Randomized Controlled Trial

**DOI:** 10.3390/nu15071792

**Published:** 2023-04-06

**Authors:** Rebecca Ahlin, Natalja P. Nørskov, Sanna Nybacka, Rikard Landberg, Viktor Skokic, Johan Stranne, Andreas Josefsson, Gunnar Steineck, Maria Hedelin

**Affiliations:** 1 Department of Oncology, Division of Clinical Cancer Epidemiology, Institute of Clinical Sciences, Sahlgrenska Academy, University of Gothenburg, 40530 Gothenburg, Sweden; rebecca.ahlin@gu.se (R.A.); viktor.skokic@ki.se (V.S.); gunnar.steineck@oncology.gu.se (G.S.); 2 Department of Animal and Veterinary Sciences, Aarhus University, AU-Foulum, 8830 Tjele, Denmark; natalja.norskov@anis.au.dk; 3 Department of Molecular and Clinical Medicine, Institute of Medicine, Sahlgrenska Academy, University of Gothenburg, 41345 Gothenburg, Sweden; sanna.nybacka@gu.se; 4 Department of Life Sciences, Division of Food and Nutrition Science, Chalmers University of Technology, 41296 Gothenburg, Sweden; rikard.landberg@chalmers.se; 5 Department of Molecular Medicine and Surgery, Karolinska Institute, 17176 Stockholm, Sweden; 6 Department of Pelvic Cancer, Karolinska University Hospital, 17176 Stockholm, Sweden; 7 Department of Urology, Institute of Clinical Sciences, Sahlgrenska Academy, University of Gothenburg, 40530 Gothenburg, Sweden; johan.stranne@vgregion.se; 8 Region Västra Götaland, Department of Urology, Sahlgrenska University Hospital, 41345 Gothenburg, Sweden; 9 Sahlgrenska Cancer Center, Department of Urology, Institute of Clinical Sciences, Sahlgrenska Academy, University of Gothenburg, 41345 Gothenburg, Sweden; andreas.josefsson@urology.gu.se; 10 Wallenberg Center for Molecular Medicine, Umeå University, 90187 Umeå, Sweden; 11 Department of Urology and Andrology, Institute of Surgery and Perioperative Sciences, Umeå University, 90187 Umeå, Sweden; 12 Regional Cancer Center West, Sahlgrenska University Hospital, Region Västra Götaland, 41345 Gothenburg, Sweden

**Keywords:** prostate cancer, phytoestrogens, isoflavones, lignans, testosterone, estradiol, sex hormone-binding globulin, insulin-like growth factor 1

## Abstract

Phytoestrogens have been suggested to have an anti-proliferative role in prostate cancer, potentially by acting through estrogen receptor beta (ERβ) and modulating several hormones. We primarily aimed to investigate the effect of a phytoestrogen intervention on hormone concentrations in blood depending on the ERβ genotype. Patients with low and intermediate-risk prostate cancer, scheduled for radical prostatectomy, were randomized to an intervention group provided with soybeans and flaxseeds (∼200 mg phytoestrogens/d) added to their diet until their surgery, or a control group that was not provided with any food items. Both groups received official dietary recommendations. Blood samples were collected at baseline and endpoint and blood concentrations of different hormones and phytoestrogens were analyzed. The phytoestrogen-rich diet did not affect serum concentrations of testosterone, insulin-like growth factor 1, or sex hormone-binding globulin (SHBG). However, we found a trend of decreased risk of increased serum concentration of estradiol in the intervention group compared to the control group but only in a specific genotype of ERβ (*p* = 0.058). In conclusion, a high daily intake of phytoestrogen-rich foods has no major effect on hormone concentrations but may lower the concentration of estradiol in patients with prostate cancer with a specific genetic upset of ERβ.

## 1. Introduction

The natural cause of prostate cancer is varying and, in some ways, poorly understood, and several hormones are believed to play a role in the prostate and the development of the disease. Firstly, androgens, especially testosterone, have been shown to play a vital function in the prostate [1]. Testosterone is converted to dihydrotestosterone (DHT) in the prostate, and transcriptional activity can be exerted by DHT binding to the androgen receptor, which is important in the progression of prostate cancer [2]. Secondly, sex hormone-binding globulin (SHBG) is a transport glycoprotein for steroid hormones with the highest affinity for androgens in the prostate [3]. Thirdly, insulin-like growth factor 1 (IGF-1) regulates the growth and development of several tissues in the body, including the prostate [4]. Nevertheless, studies have found conflicting results regarding the association between serum concentrations of testosterone [5], SHBG [5,6], and IGF-1 [4] and the development of prostate cancer. Lastly, estrogen receptor alpha and estrogen receptor beta (ERβ) have been associated with proliferative and anti-proliferative effects in prostate cancer, respectively [7].

Phytoestrogens are plant compounds with structural similarities to estrogens, especially estradiol, that can both induce or inhibit estrogenic effects due to their high binding affinity to ERβ [8,9]. By binding to the ERβ, phytoestrogens may increase prostate cancer differentiation [10,11], not only directly, but also by downregulating the androgen receptor and thus androgen-driven proliferation. Phytoestrogens are divided into three main classes: isoflavones (e.g., daidzein, genistein, glycitein), which can be found in soybeans; lignans (e.g., secoisolariciresinol, lariciresinol), which can be found in flaxseeds; and coumestans (e.g., coumestrol), which can be found in bean sprouts [8,12]. Isoflavones and lignans are metabolized by the gut microbiota [12]. Equol is formed from daidzein and secoisolariciresinol, and plant lignans are converted to the mammalian lignan enterodiol, which is subsequently transformed into enterolactone by the gut microbiota. The metabolism of phytoestrogens may depend on factors impacting the gut microbiota, e.g., the intake of antibiotics [13].

An increased intake of phytoestrogens has been associated with a decreased incidence of prostate cancer in some studies [14]. In patients with prostate cancer, several studies suggest an association between an increased intake of phytoestrogens and potentially positive effects in terms of, e.g., reduced proliferation markers [15,16,17,18]. Some studies have also found effects of phytoestrogens on hormone blood concentrations, such as testosterone, estradiol, and IGF-1 [18,19,20]. However, the results are heterogeneous, and the scientific evidence is insufficient to advise patients with prostate cancer to increase their intake of phytoestrogens [21]. In our previous case-control study, we observed that a high intake of phytoestrogens reduced the risk of prostate cancer in men with a specific polymorphic variation (TC/CC carriers) in the promoter region of ERβ [22]. These findings prompted us to investigate the hypothesis that this genotype of ERβ had a favorable effect when patients with prostate cancer increased their intake of phytoestrogens and the potential mechanism of hormones in this. Here, we primarily investigated the effect of a diet rich in phytoestrogens on hormone concentrations in blood depending on the genotype of ERβ. Secondarily, we investigated concentrations of phytoestrogens in the blood and the relationships between phytoestrogen and hormone concentrations in blood.

## 2. Materials and Methods

### 2.1. Study Population and Study Design

**Figure 1:**
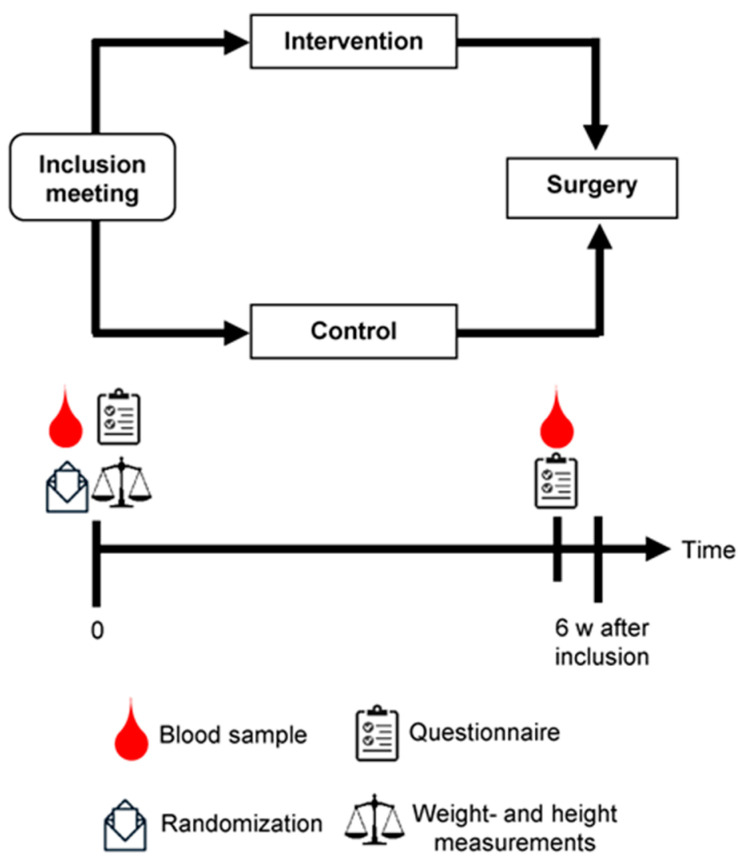
Design of the PRODICA (the impact of DIet and individual genetic factors on tumor proliferation rate in men with PROstate CAncer) study. During the inclusion meeting, participants were randomized to an intervention or a control group, filled out a questionnaire, and blood samples were collected. A similar questionnaire was filled out and blood samples were collected again within seven days before the time of surgery. The intervention was intended to last approximately 6 weeks.

The design of the PRODICA (the impact of DIet and individual genetic factors on tumor proliferation rate in men with PROstate CAncer) study and the randomization process have been described in detail elsewhere [23]. Men diagnosed with prostate cancer cT1–cT2 (prostate-specific antigen (PSA) < 20, International Society of Urological Pathology (ISUP) grade < 4) and scheduled for radical prostatectomy were invited to participate in the study at the Department of Urology at Sahlgrenska University Hospital in Gothenburg, Sweden. Patients with ongoing hormone therapy, physical or psychiatric disorders, cognitive dysfunction, and allergy to the intervention foods were not included in the study. During the inclusion meeting, the participants met a dietitian from the study administration, filled out a questionnaire, height and weight were measured, and blood samples were collected before they were randomized to an intervention or a control group (Figure 1). The study dietitian used an envelope containing folded notes, half for the intervention group and the other half for the control group, to randomize the participants. Within seven days before the time of surgery, blood samples were collected, and the participants were instructed to fill out a similar questionnaire in proximity to the time of surgery (preferably 1 to 2 days before). The study period aimed to be at least 6 weeks, but for some patients the surgery was scheduled earlier. Nevertheless, patients with at least two weeks to scheduled surgery were included in the study. However, if already included patients had surgery within two weeks after the inclusion, they were not excluded from the study. The patients were recruited between 1 February 2016, and 12 October 2022, and the last blood sample was collected in November 2022. In the PRODICA study, tumor proliferation is the primary outcome and hormone concentrations are predeclared secondary outcomes [23]. The study was registered at ClinicalTrials.gov (NCT02759380, https://clinicaltrials.gov/ct2/show/NCT02759380?cond=NCT02759380&draw=2&rank=1; accessed on 26 March 2023) on 3 May 2016 after our pilot study (*n* = 10) was finished. Except for some administrative changes, we made no major changes to the study protocol after the pilot study [23]. The study was approved by the Ethical Review Board in Gothenburg (registration number 410–14, amendment numbers T124-15; 2020-02471; 2021-03320, 2021-05878-02).

### 2.2. Intervention and Control Diets

Both groups received written dietary recommendations based on the national dietary guidelines issued by the Swedish National Food Agency [24]. The dietitian went through the guidelines orally with the participants at the inclusion meeting. The participants were instructed to avoid dietary supplements, but no other dietary restrictions were given. During the inclusion meeting, the participants in the intervention group were provided with the amounts of soybeans and flaxseeds that were planned to suffice until the scheduled surgery. The participants received a schedule on the amounts of the intervention foods to eat [23], serving suggestions, and recipes. Intake of the food items was gradually increased during the first nine days and thereafter included a daily intake of 28 g flaxseeds, 47 g green soybeans, and 28 g roasted yellow soybeans (corresponding to an estimated amount of 100 mg isoflavones and 100 mg of lignans and thus 200 mg of phytoestrogens [25]). Participants randomized to the intervention groups among the first 18 participants received crushed flaxseeds, but thereafter participants received whole flaxseeds instead due to the content of cyanogenic glycosides in flaxseeds [26], as explained in detail elsewhere [23]. Both groups were aware of which group they were allocated to, but the control group did not know what the intervention diet consisted of.

### 2.3. Blood Samples

**Figure 2:**
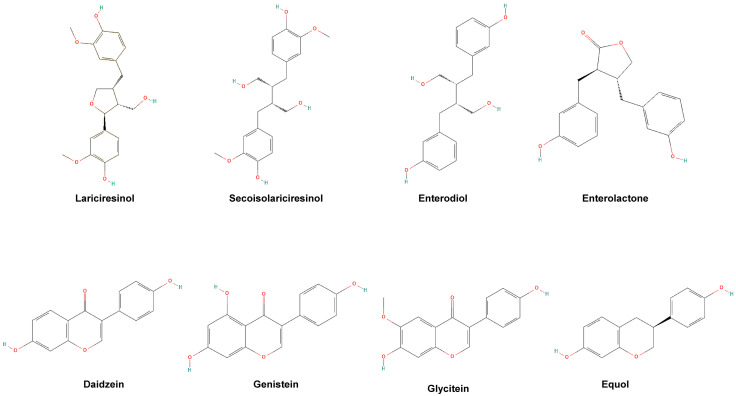
Chemical structures of the analyzed phytoestrogens in the study. Collected with permission from PubChem, URL: pubchem.ncbi.nlm.nih.gov [29].

Blood samples were collected and handled according to standard procedures [23] and were thereafter stored at −80 °C before being sent for genotyping analysis (whole blood), analysis of hormones (serum), and analysis of phytoestrogens (plasma). Analysis and selection of single nucleotide polymorphisms of the ERβ gene were performed in whole blood to assign each participant to the genotype of either TT, TC, or CC, as described elsewhere [22,23]. Plasma concentrations of phytoestrogens were analyzed at Aarhus University (Aarhus, Denmark) using LC-MS/MS measurements performed on a microLC 200 series (Eksigent/AB Sciex, Redwood City, CA, USA) and QTrap 5500 mass spectrometer (AB Sciex, Framingham, MA, USA) [27,28] with a coefficient of variation (CV) varying between 4.6% and 8.6% depending on the analyte. Quality control samples were used to calculate intra- and inter-batch CV. The chemical structures of the analyzed phytoestrogens are shown in Figure 2. The concentrations of estradiol, testosterone, SHBG, and IGF-1 were analyzed using the serum samples at the Department of Clinical Chemistry (Halland Hospital in Halmstad and Varberg, Sweden) according to their standard clinical protocol, described elsewhere [23]. However, the standard protocol for IGF-1 changed during the study period, and the first 104 participants were analyzed using sandwich enzyme-linked immunosorbent assay (ELISA), and the rest of the participants by sandwich assay on a Cobas 8000 (Hitachi High-Tech Corporation, Tokyo, Japan) analyzer series (reagent: Roche Diagnostics GmbH, Mannheim, Germany). All executors of the analyses received coded samples and were blinded to whether the samples belonged to the intervention or the control groups.

### 2.4. Statistical Analysis

Stata/SE version 17.0 (StataCorp LLC, College Station, TX, USA) was used for statistical analysis. Analyses of demographics, serum concentrations of hormones, and plasma concentrations of phytoestrogens were stratified according to the genotype of ERβ, and differences were tested between the genotypes within the intervention and control groups and between the intervention and the control groups with the same genotype. An independent t-test was used to test differences in normally distributed data and the Mann–Whitney U test or the Kruskal–Wallis test for non-normally distributed data. The Shapiro–Wilk test was used for guidance to test if the data were normally distributed. All analyses included only participants with data available from both baseline and endpoint blood samples.

To investigate changes in hormone concentrations between baseline and endpoint, we dichotomized hormone changes into increased concentrations (1) and unchanged or decreased concentrations (0) between baseline and endpoint. Then, the dichotomized variables were used as outcomes in a generalized linear model providing estimates of the risk difference (RDs) and corresponding 95% confidence intervals (CIs) of the difference between the intervention and control groups. These analyses were stratified by ERβ genotype and adjusted for body mass index (BMI), age, and smoking. Additive interactions between the ERβ genotype and intake of phytoestrogens on increased hormone concentrations were tested.

A linear regression was used to investigate the relationships between plasma concentrations of phytoestrogens (explanatory variables) and serum concentrations of hormones (outcomes). The regression model was stratified according to the intervention and control groups and adjusted for body mass index (BMI), age, and smoking. Due to skewed data, the hormone and phytoestrogen concentrations were logarithmized in the linear regression using the natural logarithm.

In the analysis of plasma concentrations of phytoestrogens, we compared users and non-users (including participants who reported “do not know”) of antibiotics over the last five years and different intervention lengths (<28, 28–56, >56 days). In a subgroup analysis, the concentrations of different lignans were stratified in participants receiving crushed and whole flaxseed. For considered confounding variables, BMI was categorized as underweight (<18.5 kg/m^2^), normal weight (18.5–24.9 kg/m^2^), overweight (25–29.9 kg/m^2^), obese class 1 (30–34.9 kg/m^2^), and obese class ≥ 2 (≥35 kg/m^2^) [30]. Age was categorized in ≥median of the study population and <median of the study population. Intake of antibiotics was categorized as 0 (non-users) if the participants reported in the questionnaire that they did not know or had no intake of antibiotics during the intervention and the recent five years. A reported intake of antibiotics, at least once during the intervention or the recent five years, was categorized as 1 (users). Smoking was categorized as current smoker (1) and nonsmoker (0). If a participant had quit smoking ≤5 years ago he was categorized as a current smoker, and if he quit smoking >5 years ago he was categorized as a nonsmoker.

## 3. Results

### 3.1. Population and Baseline Characteristics

**Figure 3:**
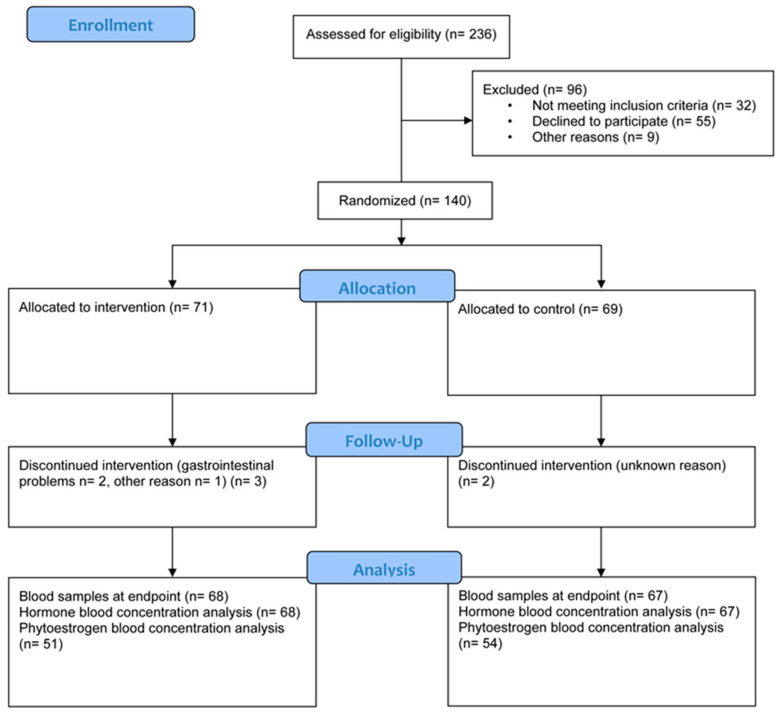
Flowchart of the PRODICA study.

Of 195 invited men, 55 patients declined to participate, with the main reasons being occupied or unwillingness to participate in the inclusion meeting (mainly due to long travel times) (Figure 3). In total, 140 participants were randomized to either the intervention (*n* = 71) or the control (*n* = 69) groups. Of these, 135 participants completed the blood sample at endpoint (intervention *n* = 68, control *n* = 67). Five participants (intervention *n* = 3, control *n* = 2) did not complete the intervention; two participants in the intervention group experienced gastrointestinal problems from the intervention foods and the participants in the control group did not state a reason. In total, seven participants in the intervention group reported gastrointestinal symptoms, and of those, five completed the intervention. Other adverse effects of the intervention foods were reported by three participants and were of different kinds.

The participants’ median age was 66 years (IQR 11; range 40–76) and the median study period was 47 days (IQR 33, range 7–583; Table 1). At the time of diagnosis, most participants had an ISUP grade of 2 and a tumor stage of T1c. At baseline, participants in the intervention group had a higher BMI, a lower level of physical activity, and a higher tumor stage, and there were less users of antibiotics in the recent years compared to participants in the control group (Table 1).

### 3.2. Effects of the Intervention Diet on Hormone Concentrations

Besides higher concentrations of estradiol at baseline in the intervention group compared to the control group in participants with the TT genotype of ERβ, there were no statistically significant differences in hormone serum concentrations between the intervention and control groups at any time points (Table 2). Within the intervention group, we found a decreased concentration of SHBG in participants with the TC/CC genotype in comparison with participants with the TT genotype who increased their concentrations. We found no effect of the intervention diet on the risk of increasing different hormone serum concentrations between baseline and endpoint, except for estradiol. There was a trend of decreased risk of increased serum concentration of estradiol in the intervention group compared to the control group but only in participants with the TC/CC genotype (RD −22%, *p* = 0.058, Table 3).

### 3.3. Plasma Concentrations of Phytoestrogens

**Figure 4:**
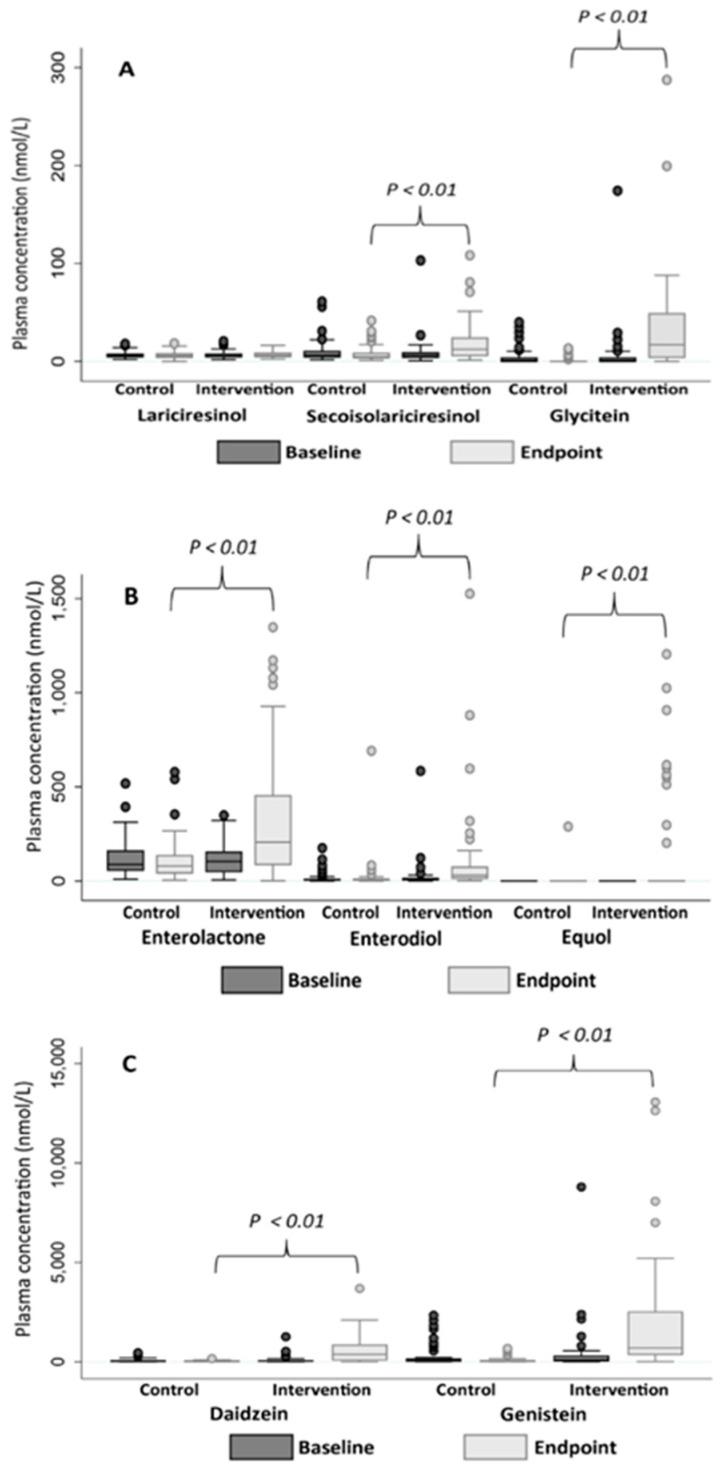
Boxplots showing plasma concentrations of different phytoestrogens (nmol/L) in the intervention- (*n* = 51) and control groups (*n* = 54) in patients with prostate cancer at baseline and endpoint. (**A**) Plasma concentrations of lariciresinol, secoisolariciresinol, and glycitein. (**B**) Plasma concentrations of enterolactone, enterodiol, and equol. (**C**) Plasma concentrations of daidzein and genistein. Concentrations of secoisolariciresinol, glycitein, enterolactone, enterodiol, equol, daidzein, and genistein were statistically significantly higher in the intervention group compared to the control group at the endpoint. The Mann–Whitney U test was used to test differences between groups.

There were no differences in the plasma concentrations of phytoestrogens at baseline between the intervention and control groups (Figure 4). The plasma concentrations of enterolactone, enterodiol, secoisolariciresinol, daidzein, genistein, glycitein, and equol were statistically significantly higher in the intervention group compared to the control group at endpoint. Participants in the intervention group increased their concentrations of these phytoestrogens during the study period compared to participants in the control group whose concentrations were maintained or reduced (Table 4). None of the participants had detectable concentrations of equol at baseline, and only ten participants (20%) in the intervention group and one participant in the control group (2%) had detectable concentrations at endpoint (Table 4).

Non-users of antibiotics had higher median values of genistein and daidzein at baseline. We found no differences in median values between users and non-users of antibiotics at endpoint or for the change between endpoint and baseline. When the change in different concentrations of phytoestrogens was compared depending on different intervention durations, no difference was found between the three different durations. Stratified analyses of participants receiving crushed and whole flaxseed showed no difference in plasma concentrations for enterodiol and enterolactone at any time point (Table S1). However, participants who received crushed flaxseeds had a higher change between baseline and endpoint in plasma concentration of secoisolariciresinol compared to those receiving whole flaxseeds.

### 3.4. The Relationship between Blood Concentrations of Phytoestrogens and Hormones

We found a relationship between higher plasma concentrations of lignans and higher serum concentrations of SHBG, but it did not remain statistically significant after adjusting for confounders. A 10% increase in plasma concentrations of lignans was associated with a 55% increase in serum concentrations of SHBG (*p* = 0.11; Table 5).

## 4. Discussion

In this randomized controlled dietary intervention study of patients with prostate cancer, an increased intake of phytoestrogens did not affect the serum of concentrations of testosterone, SHBG, and IGF-1. However, a trend of decreased risk of increased concentration of estradiol was found in participants with the TC/CC genotype of ERβ. In the intervention group, participants with the TC/CC genotype decreased serum concentrations of SHBG during the intervention compared to participants with the TT genotype, who increased their concentrations.

In one of the genotype groups of ERβ, we found a trend of a decreased risk of increasing estradiol concentration, comparing the intervention and control groups. This is in contrast to another study that demonstrated an increase in plasma concentrations of phytoestrogens and increased concentrations of serum estradiol, although the blood concentrations of phytoestrogens were lower than in our study [19]. Hamilton-Reeves et al. found higher urinary excretion of estradiol and 2-hydroxy estrogens to 16*α*-hydroxyestrone (2:16 OH-E1) ratio with an isoflavone supplement compared to a control group [31]. A higher 2:16 OH-E1 ratio has been associated with a reduced risk of prostate cancer [32]. However, 2:16 OH-E1 ratio was not analyzed in our study. A potential mechanism of our result is that the increased intake of phytoestrogens resulted in negative feedback on estradiol. To our knowledge, there is no study confirming this mechanism.

We did not find any effect of the phytoestrogen intervention on serum concentrations of testosterone, IGF-1, and SHBG. This is in line with several other studies finding no effect on blood concentrations of testosterone, IGF-1, and SHBG [15,16,17]. Other investigations challenge these results with favorable effects on testosterone [18,19] and IGF-1 in African American men [20]. These conflicting results can depend on the different doses and sources of phytoestrogens used in the studies, varying effect-modifying factors, short duration of interventions, and small sample sizes [21].

In the intervention group, the two genotype groups of ERβ had opposites effects on SHBG concentration, participants with the genotype of TC/CC decreased their concentrations during the intervention, and participants with TT genotype increased their concentrations. This suggests that the genotype of ERβ may affect the serum concentrations of hormones. To our knowledge, this is the first study to investigate the effect of phytoestrogens on serum hormones depending on the genotype of ERβ. ERβ has an important role in, e.g., hormonal and protein regulation and transcription, and thus the genotype of ERβ could play a role in these different responses [11]. Lee et al. found that postoperative biochemical recurrence-free survival was worse for patients with higher SHBG concentrations [33], suggesting a beneficial effect of the TC/CC genotype in prostate cancer. Nevertheless, the impact of the genotype of ERβ needs to be further examined in future studies.

We found that the intervention diet increased the plasma concentrations of several phytoestrogens. Elevated plasma phytoestrogen concentrations have been confirmed in similar intervention studies [19,34]. Daidzein and genistein are the main isoflavones in soybeans [12], which explains why these compounds had the highest concentrations in participants in the intervention group at endpoint. For isoflavones, few participants in our study had detectable measures of equol. Previous studies have observed a higher proportion (35%) of equol producers than ours in a general Caucasian population [35,36]. Our population’s reduced ability to metabolize equol may be caused by the fact that equol or the ability to produce equol could be related to the development of prostate cancer [37]. We noticed that the intake of antibiotics affected some phytoestrogen concentrations at baseline. This was probably because the intake of antibiotics impacted the intestinal microbiota and negatively affected the phytoestrogen metabolism [13].

The strengths of this study include the randomized design and the fact that blood concentrations of phytoestrogens were measured. The results are also based on a large clinical study with a low dropout rate. Even if the number of dropouts would be large enough to affect the results, we do not expect the results of the participants who dropped out from the study to differ from the rest of the study population. A limitation of the present study is the change from crushed to whole flaxseeds, which probably decreased the absorption of lignans [38]. This is confirmed by the higher change in concentrations of secoisolariciresinol in participants who received crushed flaxseeds compared to those who received whole flaxseeds. Another limitation is the wide range (1 to 83 weeks) of the duration of the intervention depending on when the surgery was scheduled, as both very short and very long intervention durations could have influenced the blood concentrations of phytoestrogens. However, we did not find any difference in plasma concentrations when we stratified the analysis after the intervention duration. Previous research observed half-lives of 2–11 h in plant lignans and isoflavones and longer half-lives in their mammalian conversion products, likely because of continuous transformation by the gut microbiota [39,40]. Moreover, the gut microbiota is important for the formation of enterolactone, enterodiol, and equol, but we did not collect any stool samples or information on additional factors influencing the metabolism of phytoestrogens (e.g., diseases or drugs influencing gut microbiota and intake of prebiotics and probiotics) [12,41]. For the generalizability of the result, we lack data on patients with high-grade prostate cancer, men without prostate cancer, and women.

In conclusion, our findings suggest that a high intake of phytoestrogens may lower the concentration of estradiol in patients with prostate cancer with a specific genetic upset of ERβ but does not affect serum concentrations of testosterone, IGF-1, and SHBG. However, the effect on SHBG concentration differed across the ERβ genotype groups. The effect of the genotype of ERβ on hormone concentrations in patients with prostate cancer should be confirmed in future studies. Further research is needed to investigate whether elevated plasma concentrations of phytoestrogens have a beneficial effect in terms of reduced tumor proliferation and prolonged survival for patients with prostate cancer.  

## Figures and Tables

**Table 1:** Demographics of the patients included in the PRODICA ^1^ study.

	Intervention (*n* = 68)				Control (*n* = 67)			
	Genotype TT ^2^ (*n* = 35)		Genotype TC/CC ^2^ (*n* = 33)		Genotype TT ^2^ (*n* = 26)		Genotype TC/CC ^2^ (*n* = 41)	
	Median (IQR)	Range	Median (IQR)	Range	Median (IQR)	Range	Median (IQR)	Range
Age, years	65 (13)	51–76	67 (8)	43–76	66 (10)	51–74	65 (10)	40–75
Intervention period, d	47 (46)	12–189	48 (28)	7–146	46 (27)	8–213	47 (29)	14–583
BMI, kg/m^2^	27.8 (5.2)	21.7–37.4	28.1 (4.7)	21.3–35.1	26.0 (3.9)	20.0–40.0	25.5 (3.8)	20.6–33.3
	***n*** **(%)**		***n*** **(%)**		***n*** **(%)**		***n*** **(%)**	
**Tumor stage at diagnosis**								
cT1	20 (57)		20 (61)		14 (54)		31 (76)	
cT2	15 (43)		12 (36)		10 (38)		10 (24)	
cTX	0 (0)		1 (3)		2 (8)		0 (0)	
**ISUP grade at diagnosis**								
1	11 (31)		14 (42)		10 (38)		19 (46)	
2	19 (54)		15 (45)		11 (42)		19 (46)	
3	5 (14)		4 (12)		5 (19)		3 (7)	
**Physical activity ^3^**								
Low	6 (17)		8 (24)		2 (8)		4 (10)	
Moderate	19 (54)		16 (48)		17 (65)		19 (46)	
High	10 (29)		9 (27)		7 (27)		18 (44)	
**Heredity**								
Yes	13 (37)		12 (36)		7 (27)		14 (34)	
No	9 (26)		10 (30)		4 (15)		14 (34)	
Do not know	13 (37)		11 (33)		15 (58)		13 (32)	
**Antibiotic treatment last year**								
Yes	12 (34)		7 (21)		11 (42)		11 (27)	
No	22 (63)		25 (76)		15 (58)		29 (71)	
Do not know	1 (3)		1 (3)		0 (0)		1 (2)	
**Antibiotic treatment last 2–5 years**								
Yes	14 (40)		10 (30)		13 (50)		22 (54)	
No	19 (54)		17 (52)		12 (46)		14 (34)	
Do not know	2 (6)		6 (18)		1 (4)		5 (12)	
**Antibiotic treatment during the** **intervention, *n* (%)**								
Yes	1 (3)		3 (9)		3 (12)		4 (10)	
No	34 (97)		30 (91)		23 (88)		36 (88)	
Missing, *n* (%)	0 (0)		0 (0)		0 (0)		1 (2)	
**Smoking**								
Currently	2 (6)		1 (3)		1 (4)		2 (5)	
Previously	16 (46)		17 (52)		12 (46)		21 (51)	
Never	17 (49)		15 (45)		13 (50)		18 (44)	

^1^ The impact of DIet and individual genetic factors on tumor proliferation rate in men with PROstate CAncer. ^2^ Participants were assigned to the genotype TT, TC, or CC of the estrogen receptor beta. ^3^ Activity in the daytime: sedentary (100 p); partly sedentary, sitting, and walking (200 p); mostly standing and walking (300 p), physical labor (400 p). Physical activity in the evening time: sedentary (1 p), slight activity—equal to a 30-min walk (2 p); moderately strenuous activity—equal to a bike ride of ≥30 min (3 p); sports activity (4 p). Low physical activity: 101–103, 201 p; moderate physical activity: 104, 202–203, 301–302 p; high physical activity: 204, 303–304, 401–404 p. Abbreviations: ISUP, International Society of Urological Pathology.

**Table 2:** Serum concentrations of hormones in the intervention and control groups in patients with prostate cancer, stratified by the genotype of estrogen receptor beta.

	Intervention (*n* = 68)				Control (*n* = 67)						*p * ^1^	
	Genotype TT ^2^ (*n* = 35)		Genotype TC/CC ^2^ (*n* = 33)			Genotype TT ^2^ (*n* = 26)		Genotype TC/CC ^2^ (*n* = 41)			TT	TC/CC
	Median (IQR)	Range	Median (IQR)	Range	*p * ^3^	Median (IQR)	Range	Median (IQR)	Range	*p * ^3^		
**Testosterone (nmol/L)**												
Baseline	15.3 (6.6)	9.6–30.1	15.0 (6.4)	5.1–26.2	0.710 ^5^	15.6 (6.3)	8.0–22.7	14.9 (7.5)	8.4–32.6	0.889	0.521 ^5^	0.699 ^5^
Endpoint	14.8 (4.9)	6.9–22.6	14.2 (6.4)	5.1–30.0	0.566 ^5^	14.4 (6.7)	7.3–26.0	15.8 (8.8)	5.4–30.5	0.331 ^5^	0.483 ^5^	0.362 ^5^
Change ^4^	−0.5 (3.7)	−10.1–13.0	0.2 (4.8)	−9.0–9.0	0.854 ^5^	−0.8 (2.0)	−8.1–9.6	−0.6 (3.8)	−7.7–18.3	0.648	0.814	0.865
**Estradiol (nmol/L)**												
Baseline	0.099 (0.034)	0.069–0.18	0.10 (0.029)	0.040–0.18	0.273	0.090 (0.040)	0.049–0.15	0.092 (0.040)	0.030–0.32	0.500	0.0137 ^5^	0.546
Endpoint	0.10 (0.048)	0.045–0.18	0.10 (0.034)	0.048–0.16	0.973^5^	0.088 (0.035)	0.023–0.15	0.10 (0.061)	0.033–0.35	0.149	0.0625 ^5^	0.727
Change ^4^	−0.0050 (0.027)	−0.050–0.055	0.0 (0.026)	−0.038–0.048	0.0725	−0.0015 (0.027)	−0.026–0.034	0.0050 (0.032)	−0.046–0.089	0.159	0.679 ^5^	0.657 ^5^
**SHBG (nmol/L)**												
Baseline	49.0 (25.0)	28.0–114.0	45.0 (25.0)	21.0–110.0	0.640	50.0 (22.0)	19.0–94.0	48.0 (32.0)	22.0–126.0	0.947	0.848	0.744
Endpoint	51.0 (22.0)	28.0–113.0	41.0 (22.0)	20.0–96.0	0.0982	50.5 (29.0)	24.0–96.0	51.0 (33.0)	20.0–112.0	0.797	0.980	0.273
Change ^4^	1.0 (8.0)	−25.0–16.0	−2.0 (10.0)	−29.0–13.0	0.00390	0.0 (6.0)	−12.0–21.0	−2.0 (8.0)	−20.0–25.0	0.189	0.502	0.418
**IGF** **−1 (µg/L)**												
Baseline	126.0 (62.0)	75.0–280.0	138.0 (56.0)	60.0–276.0	0.915	166.5 (44.0)	90.0–274.0	146.0 (64.0)	68.0–381.0	0.173	0.0973	0.536
Endpoint	135.0 (57.0)	82.0–304.0	151.0 (64.0)	59.0–266.0	0.819	161.0 (81.0)	90.0–253.0	153.0 (85.0)	63.0–340.0	0.864	0.301	0.499 ^5^
Change ^4^	−1.0 (18.0)	−25.0–124.0	7.0 (23.0)	−66.0–41.0	0.281	−0.5 (26.0)	−65.0–54.0	7.0 (31.0)	−45.0–55.0	0.232	0.656	0.955 ^5^
**Testosterone/SHBG ratio**												
Baseline	0.33 (0.10)	0.14–0.55	0.32 (0.080)	0.16–0.49	0.826 ^5^	0.30 (0.099)	0.18–0.67	0.32 (0.12)	0.17–0.56	0.426	0.319	0.828 ^5^
Endpoint	0.30 (0.12)	0.17–0.58	0.33 (0.12)	0.19–0.59	0.215 ^5^	0.28 (0.11)	0.17–0.47	0.32 (0.12)	0.17–0.60	0.0705	0.380	0.773
Change ^4^	0.011 (0.090)	−0.19–0.17	−0.0023 (0.088)	−0.12–0.094	0.0734 ^5^	0.012 (0.068)	−0.092–0.26	0.00043 (0.067)	−0.29–0.12	0.145	0.789	0.462
**Testosterone/estradiol ratio**												
Baseline	134.9 (37.4)	0.09–311.1	140.5 (77.8)	0.09–348.5	0.637	166.1 (75.6)	0.1–346.2	155.0 (12.6)	0.09–380.0	0.280	0.0697	0.854
Endpoint	149.3 (69.9)	0.1–322.2	140.4 (84.0)	0.083–321.7	0.678	164.1 (102.1)	0.1–487.0	138.8 (127.1)	0.09–356.4	0.242	0.107	0.681
Change ^4^	0.07 (46.4)	−70.1–68.9	−6.4 (40.7)	−95.0–54.6	0.127 ^5^	−5.2 (48.4)	−106.3–225.7	−2.8 (26.1)	−116.4–108.3	0.934	0.438	0.417

^1^ Difference between the intervention and control groups within the same genotype of the estrogen receptor beta. ^2^ Participants were assigned to the genotype of either TT, TC, or CC of the estrogen receptor beta. ^3^ Difference between genotypes within the intervention and control groups. ^4^ The median difference between endpoint and baseline. ^5^ An independent T-test test was used to compare groups depending on the normal distribution. The Mann–Whitney U test was used to compare groups, except when noted otherwise. Abbreviations: IGF-1, insulin-like growth factor 1; SHBG, sex hormone binding globulin.

**Table 3:** Risk differences (RDs) with 95% confidence intervals (CIs) for the risk of increasing different hormone concentrations between baseline and endpoint, in relation to intake of phytoestrogens, stratified by estrogen receptor beta genotype (TT or TC/CC).

Hormone Concentrations (nmol/L)		RD	95% CI	Adjusted ^1^ RD	Adjusted ^1^ 95% CI	*p* Additive Interaction
**Testosterone**	All cases (*n* = 135)	0.083	−0.84, 0.25	0.067	−0.10, 0.23	0.792
	TT (*n* = 61)	0.12	−0.12, 0.36	0.099	−0.14, 0.34	
	TC/CC (*n* = 74)	0.076	−0.15, 0.30	0.073	−0.16, 0.31	
**Estradiol**	All cases (*n* = 135)	−0.11	−0.28, 0.057	−0.13	−0.30, 0.045	0.424
	TT (*n* = 61)	−0.013	−0.26, 0.23	−0.027	−0.27, 0.22	
	TC/CC (*n* = 74)	−0.15	−0.37, 0.076	−0.22	−0.45, 0.071	
**SHBG**	All cases (*n* = 135)	0.038	−0.13, 0.20	0.030	−0.14, 0.20	0.149
	TT (*n* = 61)	0.15	−0.10, 0.40	0.13	−0.12, 0.37	
	TC/CC (*n* = 74)	−0.093	−0.30, 0.12	−0.11	−0.33, 0.011	
**IGF-1**	All cases (*n* = 135)	−0.038	−0.21, 0.13	−0.028	−0.20, 0.14	0.386
	TT (*n* = 61)	−0.090	−0.34, 0.16	−0.076	−0.31, 0.15	
	TC/CC (*n* = 74)	0.057	−0.16, 0.28	0.068	−0.16, 0.30	
**Testosterone/SHBG ratio**	All cases (*n* = 135)	0.0061	−0.16, 0.17	0.027	−0.14, 0.20	0.632
	TT (*n* = 61)	0.022	−0.21, 0.25	0.028	−0.20, 0.25	
	TC/CC (*n* = 74)	−0.058	−0.29, 0.17	−0.083	−0.31, 0.15	
**Testosterone/estradiol ratio**	All cases (*n* = 135)	0.097	−0.068, 0.26	0.081	−0.090, 0.25	0.458
	TT (*n* = 61)	0.15	−0.10, 0.40	0.13	−0.12, 0.39	
	TC/CC (*n* = 74)	0.022	−0.20, 0.24	−0.0050 ^2^ 0.0011 ^3^ 0.024 ^4^	−0.21, 0.22 ^2^ −0.21, 0.21 ^3^ −0.21, 0.25 ^4^	

^1^ Analyses were adjusted for BMI (kg/m2) (≤18.5; 18.5 to <25; 25 to <30; 30 to <35; ≥35), age (≥median, <median), and smoking (1 = current smoker or quit smoking ≤5 years ago; 0 = nonsmoker or quit smoking >5 years ago). ^2^ The analysis did not converge and was therefore only adjusted for BMI and smoking. ^3^ The analysis did not converge and was therefore only adjusted for age and smoking. ^4^ The analysis did not converge and was therefore only adjusted for BMI and age. Abbreviations: IGF-1, insulin-like growth factor 1; SHBG, sex hormone binding globulin.

**Table 4:** Plasma concentrations of phytoestrogens in the intervention and control groups in patients with prostate cancer, stratified by the genotype of estrogen receptor beta.

	Intervention (*n* = 51)					Control (*n* = 54)					*p * ^1^	
	Genotype TT ^2^ (*n* = 26)		Genotype TC/CC ^2^ (*n* = 25)			Genotype TT ^2^ (*n* = 24)		Genotype TC/CC ^2^ (*n* = 30)			TT	TC/CC
Plasma Concentrations (nmol/L)	Median (IQR)	Range	Median (IQR)	Range	*p * ^3^	Median (IQR)	Range	Median (IQR)	Range	*p * ^3^		
**Lariciresinol**												
Baseline	4.9 (4.1)	1.8–17.0	5.2 (4.2)	1.7–20.6	0.797	5.9 (2.5)	3.1–12.2	5.7 (6.5)	2.0–17.9	0.833	0.718	0.654
Endpoint	6.5 (4.9)	2.6–12.0	6.0 (6.0)	2.4–16.4	0.374	6.1 (5.5)	1.2–18.4	4.6 (4.9)	0.0–15.6	0.419	0.473	0.463
Change ^4^	1.9 (5.8)	−11.0–8.5	−0.02 (4.3)	−10.4–7.1	0.449	0.02 (5.8)	−6.7–11.8	−1.3 (5.1)	−12.4–5.3	0.138	0.481	0.164
**Secoisolariciresinol**												
Baseline	6.5 (7.1)	0.8–26.7	5.7 (5.5)	0.6–103.1	0.664	6.0 (8.6)	1.5–61.1	6.2 (6.9)	1.8–31.0	0.883	0.859	0.507
Endpoint	13.9 (17.9)	2.2–80.8	10.4 (9.9)	1.2–108.3	0.275	5.5 (11.0)	1.8–29.6	4.8 (4.5)	0.9–41.6	0.766	0.0094	0.0314
Change ^4^	5.0 (21.4)	−17.2–78.5	4.2 (11.1)	−90.3–98.5	0.647	0.08 (6.5)	−50.1–26.8	−0.8 (5.2)	−25.4–18.9	0.506	0.0068	0.0045
**Enterodiol**												
Baseline	7.0 (10.2)	0.8–122.0	5.7 (15.1)	0.6–584.5	0.507	7.7 (23.0)	1.2–175.0	4.5 (6.9)	0.4–81.9	0.0932	0.683	0.694
Endpoint	25.5 (37.5)	4.3–880.3	32.0 (122.4)	1.2–1526.1	0.604	8.8 (11.6)	0.5–691.3	4.5 (9.4)	0.2–82.6	0.244	<0.001	<0.001
Change ^3^	11.7 (32.6)	−87.9–876.8	17.1 (92.3)	−330.8–1501.2	0.395	−1.6 (14.6)	−114.1–683.5	−0.2 (5.7)	−52.0–77.4	0.264	0.0014	0.0014
**Enterolactone**												
Baseline	113.6 (123.0)	10.7–348.7	71.2 (107.5)	5.2–321.8	0.207	107.4 (144.3)	8.6–517.7	66.0 (69.0)	8.4–393.7	0.115	0.981	0.923
Endpoint	213.0 (404.8)	0.8–1348.2	175.4 (371.7)	5.3–1172.1	0.344	78.8 (123.6)	4.5–578.8	78.1 (91.0)	15.8–231.9	0.572	<0.001	0.0048
Change ^4^	109.1 (364.4)	−218.2–1264.5	109.1 (270.7)	−215.0–1052.0	0.993	−29.2 (92.9)	−168.3–446.0	−22.2 (61.9)	−213.9–203.8	0.714	0.0013	<0.001
**Daidzein**												
Baseline	40.3 (112.1)	0.0–477.8	32.4 (53.0)	0.0–1257.5	0.426	16.4 (46.7)	0.0–349.0	33.3 (94.7)	0.0–453.0	0.776	0.372	0.792
Endpoint	579.2 (796.6)	0.0–2096.7	250.0 (819.4)	0.0–3688.4	0.384	10.2 (23.4)	0.0–153.0	32.3 (38.3)	0.0–157.7	0.124	<0.001	<0.001
Change ^4^	499.1 (790.5)	−205.0–2093.2	132.3 (680.6)	−89.1–3655.4	0.472	−7.6 (33.5)	−308.9–135.7	−3.2 (66.9)	−365.9–142.5	0.554	<0.001	<0.001
**Genistein**												
Baseline	147.5 (325.1)	0.0–8798.1	68.1 (163.4)	0.0–2378.8	0.486	34.3 (150.6)	0.0–2335.0	43.8 (157.1)	0.0–2091.8	0.675	0.290	0.782
Endpoint	1055.5 (3141.7)	4.4–8075.1	474.9 (1378.7)	10.3–13,070.3	0.129	18.5 (127.4)	0.0–667.7	29.3 (79.9)	0.0–621.7	0.382	<0.001	<0.001
Change ^4^	668.6 (3003.5)	−5060.9–8041.4	421.7 (692.8)	−322.1–12,911.8	0.438	−13.6 (144.4)	−1667.3–201.1	−14.2 (86.5)	−1470.1–438.2	0.982	<0.001	<0.001
**Glycitein**												
Baseline	0.0 (4.4)	0.0–29.2	0.0 (3.4)	0.0–174.3	0.853	0.0 (3.3)	0.0–29.6	0.0 (4.4)	0.0–40.0	0.905	0.834	0.678
Endpoint	20.9 (43.3)	0.0–87.9	14.4 (42.7)	0.0–287.4	0.406	0.0 (0.0)	0.0–6.1	0.0 (2.2)	0.0–13.2	0.130	<0.001	<0.001
Change ^4^	17.3 (47.5)	−10.9–83.7	14.4 (40.0)	−5.0–287.4	0.604	0.0 (3.3)	−25.0–3.5	0.0 (4.1)	−40.0–13.2	0.816	<0.001	<0.001
**Equol**												
Baseline	0.0 (0.0)	0.0–0.0	0.0 (0.0)	0.0–0.0	1.000	0.0 (0.0)	0.0–0.0	0.0 (0.0)	0.0–0.0	1.000	1.000	1.000
Endpoint	0.0 (0.0)	0.0–1204.9	0.0 (0.0)	0.0–616.1	0.404	0.0 (0.0)	0.0–0.0	0.0 (0.0)	0.0–289.0	1.000	0.0290	0.0742
Change ^4^	0.0 (0.0)	0.0–1204.9	0.0 (0.0)	0.0–616.1	0.404	0.0 (0.0)	0.0–0.0	0.0 (0.0)	0.0–289.0	1.000	0.0290	0.0742

^1^ Difference between the intervention and control groups within the same genotype of the estrogen receptor beta. ^2^ Participants were assigned to the genotype of either TT, TC, or CC of the estrogen receptor beta. ^3^ Difference between genotypes of the estrogen receptor beta within the intervention- and control groups. ^4^ The median difference between endpoint and baseline.

**Table 5:** Linear regression analyses between plasma concentrations of phytoestrogens (explanatory variables) and serum concentrations of hormones (outcomes) in patients with prostate cancer (*n* = 105).

Hormone Concentrations (nmol/L)		Plasma Concentrations of Lignans ^1^ (nmol/L) β (95% CI)	Plasma Concentrations of Isoflavones ^2^ (nmol/L) β (95% CI)	Plasma Concentrations of Phytoestrogens ^3^ (nmol/L) β (95% CI)
**Testosterone**	Unadjusted	0.035 (−0.028, 0.099)	0.0071 (−0.024, 0.039) ^4^	0.013 (−0.034, 0.059)
	Adjusted ^5^	0.029 (−0.034, 0.092)	0.0099 (−0.021, 0.041) ^4^	0.014 (−0.031, 0.059)
**Estradiol**	Unadjusted	0.019 (−0.051, 0.089)	−0.012 (−0.046, 0.022) ^4^	−0.013 (−0.064, 0.037)
	Adjusted ^5^	0.026 (−0.044, 0.097)	−0.015 (−0.050, 0.019) ^4^	−0.014 (−0.65, 0.036)
**SHBG**	Unadjusted	0.071 (0.0013, 0.14)	−0.0090 (−0.044, 0.026) ^4^	0.011 (−0.040, 0.062)
	Adjusted ^5^	0.055 (−0.013, 0.12)	−0.0048 (−0.038, 0.029) ^4^	0.010 (−0.039, 0.059)
**IGF-1**	Unadjusted	0.0065 (−0.049, 0.062)	0.012 (−0.015, 0.038) ^4^	0.0085 (−0.031, 0.048)
	Adjusted ^5^	0.015 (−0.041, 0.071)	0.0095 (−0.017, 0.036) ^4^	0.0093 (−0.031, 0.049)
**Testosterone/SHBG ratio**	Unadjusted	−0.036 (−0.85, 0.014)	0.016 (−0.0082, 0.040) ^4^	0.0017 (−0.034, 0.038)
	Adjusted ^5^	−0.026 (−0.075, 0.023)	0.015 (−0.0093, 0.039) ^4^	0.0038 (−0.031, 0.039)
**Testosterone/estradiol ratio**	Unadjusted	0.016 (−0.057, 0.089)	0.019 (−0.016, 0.055) ^4^	0.026 (−0.026, 0.078)
	Adjusted ^5^	0.0031 (−0.064, 0.070)	0.025 (−0.0071, 0.058) ^4^	0.028 (−0.019, 0.076)

^1^ Include lariciresinol, secoisolariciresinol enterolactone, and enterodiol. ^2^ Include daidzein, genistein, glycitein, and equol. ^3^ Include isoflavones and lignans. ^4^ One participant is missing. ^5^ Analyses were adjusted for BMI (kg/m2) (≤18.5; 18.5 to <25; 25 to <30; 30 to <35; ≥35), age (≥median, <median), and smoking (1 = current smoker or quit smoking ≤5 years ago; 0 = nonsmoker or quit smoking >5 years ago). Samples of plasma and serum were collected at endpoint. Hormone concentrations and phytoestrogen concentrations were logarithmized using the natural logarithm. Abbreviations: β, beta-coefficient; IGF-1, insulin-like growth factor 1; SHBG, sex hormone binding globulin.

## Data Availability

The data presented in this study are available on request from the corresponding author. The data are not publicly available due to analyses that are ongoing for future publications.

## Supplementary Materials

The following supporting information can be downloaded at: https://www.mdpi.com/article/10.3390/nu15071792/s1, Table S1: Plasma concentrations of phytoestrogens in the intervention group receiving crushed or whole flaxseeds in the PRODICA study.
