# Supplementary material for: Effects on Serum Hormone Concentrations after a Dietary Phytoestrogen Intervention in Patients with Prostate Cancer: A Randomized Controlled Trial
Source: Nutrients. 2023 Apr 6;15(7):1792. doi: 10.3390/nu15071792 (PMC10097251; doi:10.3390/nu15071792)
Supplement: Supplementary file 1 [file nutrients-15-01792-s001.zip › nutrients-2305265-supplementary.pdf]

Supplementary Materials

Supplemental Table S1. Plasma concentrations of phytoestrogens in the intervention group receiving crushed or whole flaxseeds in the PRODICA [1] study

	**Crushed flaxseeds (*n* = 7)**		**Whole flaxseeds (*n* = 44)**		
**Plasma concentrations (nmol/L)**	**Median (IQR)**	**Range**	**Median (IQR)**	**Range**	***P***
**Secoisolariciresinol**					
Baseline	7.5 (9.0)	1.3-14.8	6.0 (5.6)	0.6-103.1	1.000
Endpoint	36.7 (39.0)	7.8-80.8	11.5 (17.2)	1.2-108.3	0.0552
Change [2]	25.4 (32.7)	1.0.2-78.4	4.2 (12.7)	-90.3-98.5	0.0342
**Enterodiol**					
Baseline	5.6 (19.0)	1.1-1584.5	5.9 (11.4)	0.6-122.0	0.989
Endpoint	29.2 (98.6)	5.4-253.8	30.0 (58.1)	1.2-1526.1	0.943
Change [2]	9.1 (105.7)	-330.8-100.5	16.6 (53.6)	-87.9-1501.2	0.412
**Enterolactone**					
Baseline	117.2 (205.3)	46.6-321.8	87.4 (113.0)	5.2-348.7	0.253
Endpoint	438.6 (771.4)	66.9-1042.6	204.0 (368.2)	0.8-1348.2	0.312
Change[2]	116.8 (718.0)	6.1-779.3	102.0 (355.2)	-218.2-1264.5	0.299

[1] The impact of DIet and individual genetic factors on tumor proliferation rate in men with PROstate CAncer.
[2] The median difference between endpoint and baseline.
The Mann-Whitney U test was used to compare groups.
